# Sex-different hepaticglycogen content and glucose output in rats

**DOI:** 10.1186/1471-2091-11-38

**Published:** 2010-09-23

**Authors:** Carolina Gustavsson, Kamal Yassin, Erik Wahlström, Louisa Cheung, Johan Lindberg, Kerstin Brismar, Claes-Göran Östenson, Gunnar Norstedt, Petra Tollet-Egnell

**Affiliations:** 1 Department of Molecular Medicine and Surgery, Karolinska Institutet, Sweden; 2 Molecular Toxicology, Safety Assessment, Astra Zeneca R&D, Sweden

## Abstract

**Background:**

Genes involved in hepatic metabolism have a sex-different expression in rodents. To test whether male and female rat livers differ regarding lipid and carbohydrate metabolism, whole-genome transcript profiles were generated and these were complemented by measurements of hepatic lipid and glycogen content, fatty acid (FA) oxidation rates and hepatic glucose output (HGO). The latter was determined in perfusates from *in situ *perfusion of male and female rat livers. These perfusates were also analysed using nuclear magnetic resonance (NMR) spectroscopy to identify putative sex-differences in other liver-derived metabolites. Effects of insulin were monitored by analysis of Akt-phosphorylation, gene expression and HGO after s.c. insulin injections.

**Results:**

Out of approximately 3 500 gene products being detected in liver, 11% were significantly higher in females, and 11% were higher in males. Many transcripts for the production of triglycerides (TG), cholesterol and VLDL particles were female-predominant, whereas genes for FA oxidation, gluconeogenesis and glycogen synthesis were male-predominant. Sex-differences in mRNA levels related to metabolism were more pronounced during mild starvation (12 h fasting), as compared to the postabsorptive state (4 h fasting). No sex-differences were observed regarding hepatic TG content, FA oxidation rates or blood levels of ketone bodies or glucose. However, males had higher hepatic glycogen content and higher HGO, as well as higher ratios of insulin to glucagon levels. Based on NMR spectroscopy, liver-derived lactate was also higher in males. HGO was inhibited by insulin in parallel with increased phosphorylation of Akt, without any sex-differences in insulin sensitivity. However, the degree of Thr172-phosphorylated AMP kinase (AMPK) was higher in females, indicating a higher degree of AMPK-dependent actions.

**Conclusions:**

Taken together, males had higher ratios of insulin to glucagon levels, higher levels of glycogen, lower degree of AMPK phosphorylation, higher expression of gluconeogenic genes and higher hepatic glucose output. Possibly these sex-differences reflect a higher ability for the healthy male rat liver to respond to increased energy demands.

## Background

In most mammalian species post-pubertal growth, body size and body composition are sexually dimorphic [1]. Muscle mass (especially upper body muscle mass) is greater in males than females, whereas women generally have a higher amount of body fat [2]. When obesity develops, women have a higher proportion of body fat in the gluteal-femoral region, whereas men have more body fat in the abdominal (visceral) region [3]. Since visceral obesity is strongly correlated to the development of obesity-related diseases (hyperlipidemia, hypertension, type-2 diabetes and cardiovascular disease), differences in body composition might explain why the metabolic syndrome is greater for men than age-matched premenopausal women [4-6]. Other sex-dependent factors that may contribute to a more insulin-sensitive environment in women than in men include sex hormones and adipokines [7]. In addition, nonalcoholic fatty liver disease (NAFLD) is closely associated with metabolic disorders [8], and it has been shown that male gender, waist circumference, triglyceride level, and insulin resistance are independently associated with NAFLD in non-obese subjects [9].

The prevalence of early abnormalities of glucose metabolism has been estimated to be higher in men than in premenopausal women [10]. Since women develop the metabolic syndrome approximately ten years later than men, it has been suggested that the endocrine status in fertile women might be protective in this context. This has been supported by data from animal studies showing that ERKO (estrogen receptor α knockout) mice develop fatty liver, hepatic insulin resistance and impaired glucose tolerance [11]. This indicates that down-stream effects of sex-dependent hormones within the liver might play a role during development of NAFLD. Improved insight regarding sex-differences in hepatic lipid and carbohydrate homeostasis may therefore facilitate attempts to unravel the mechanisms behind metabolic disease development.

To gain a deeper insight into the sex-differentiated activities of the liver, we [12] and others [13,14] have previously used transcript profiling to identify gene products with sex-different expression levels. Although these studies differ regarding design, it was jointly found that genes involved in lipid, steroid and drug metabolism have a sex-different expression in rodent liver. It seems likely that sets of genes from specific metabolic pathways are regulated in a coordinated manner and that sex-dependent hormones have an important impact on the long-term regulation of these genes. However, since differences observed at the mRNA level are not always translated into the same differences at the level of protein function, transcript data need to be complemented by functional studies.

The liver has a major role in energy storage after a meal, as well as in the release of fuel molecules during situations such as starvation. Based on previous studies in both humans and animals [15], males might be predicted to more rapidly mobilize hepatic carbohydrates as compared to females. Males are also more responsive to peroxisome proliferators than female rats [16-19], including higher rates of lipid oxidation. It might thus be speculated that male livers have a greater production of reactive oxygen species (ROS) during situations of increased hepatic fat. NAFLD is believed to be caused by fat accumulation in the liver and through increased ROS production lead to hepatic insulin resistance [20]. Female livers have been shown to be more efficient in packaging long chain fatty acids into very-low-density-lipoprotein (VLDL) particles [21], which might reduce the hepatocellular load of lipids. Taken together, these differences might possibly contribute to a higher risk of developing hyperglycemia and hepatic insulin resistance in males as compared to females during situations of increased hepatic fat and/or insufficient insulin production.

Glycogen storage, release of glucose and ketone bodies are major functions of the liver that differ depending on the metabolic state of the individual. In this study we aimed to substantiate the hypothesis that healthy (lean) male and female rat livers differ regarding these functions. We believe that this could provide a basis for an improved understanding of which mechanisms that might be involved in the development of sex-different disease linked to the liver. Male and female rats, fed standard diet and fasted for either 4 h or 12 h, were compared regarding hepatic gene expression, lipid and glycogen content, fatty acid (FA) oxidation rate, release of glucose and ketone bodies. Furthermore, liver perfusates were analysed using nuclear magnetic resonance (NMR) spectroscopy to identify putative sex-differences in other liver-derived metabolites.

## Methods

### Animals

#### Exp 1

Sex-dependent effects on hepatic gene expression in non-fasted male and female rats: Seven-week-old male (n = 5) and female (n = 5) Sprague-Dawley (SD) rats (Scanbur BK) were maintained under standardized conditions with free access to regular rodent chow (R36, Lactamin, Sweden) and water. Rats were sacrificed around noon, livers removed and frozen in liquid nitrogen. These livers were used for transcript profiling (whole genome microarray analysis) only.

#### Exp 2

Effects of 4 h and 12 h fasting in male and female rats: Seven-week-old male (n = 16) and female (n = 16) SD rats (Scanbur BK) were maintained as described above. Food was removed early in the morning (7 a.m.) or late in the evening (11 p.m.), so that the animals were without food for either 4 h (absorptive state) or 12 h (post-absorptive state). Body weights were not significantly altered by this short period of food deprivation (4 h males 255.3 ± 6.6 g; 12 h males 238.8 ± 6.6 g; 4 h females 213.3 ± 5.2 g; 12 h females 201.3 ± 3.1 g).

To determine rapid hepatic insulin responses, rats were injected intraperitoneally (i.p.) with insulin (Actrapid, Novo Nordisk), at a dose of 5 mU/g body weight, or saline only. After 40 minutes of treatment rats were sacrificed, blood drawn from vena cava, livers removed and frozen in liquid nitrogen. As described below, separate animals (n = 32) were used for measurements of hepatic glucose output (since perfused livers are unsuitable for analyses such as gene expression). All animal experiments were approved by the regional Ethics Committee on Animal Experiments.

### Plasma and blood analysis

Blood was collected from vena cava in 10-mL tubes containing 17.5 mg EDTA (Becton, Dickinson and Company). Plasma was obtained by centrifugation at 3000 g for 10 min. The resulting supernatants were removed and analysed for insulin, glucagon (RIA kits from Millipore), corticosterone (EIA kit from Diagnostic Systems Laboratories). Levels of blood glucose and ketone bodies were anlysed in a drop of blood collected from the tip of the tail using a Precision Xtra glucometer and test strips (Abbot Scandinavia).

### Expression profiling using microarrays

Microarrays containing 70 mer oligonucleotide probes for 27 649 rat protein-coding genes were fabricated and used to obtain transcript profiles, as described previously [22]. Each hybridisation compared Cy3-labeled cDNA reverse transcribed from RNA isolated from female rat livers with Cy5-labeled cDNA isolated from male livers. Each experiment was analysed using individual samples and dye-swopping. Identification of sex-dependent genes was performed using the SAM 1.21 (Significance Analysis for Microarray) software incorporated in Microsoft office Excel program. A 5% false discovery rate was used as a first cut-off. Genes with a greater than 1.5-fold difference between the sexes were considered as being sex-dependent [12]. The results are represented as the mean of at least three independent determinations. All data are available from the NCBI Gene Expression Omnibus database (GEO; http://www.ncbi.nlm.nih.gov/geo/) using the series entry GSE20601.

### Analysis of gene expression by real time quantitative RT-PCR

Total hepatic RNA was isolated, cDNA generated and gene expression quantified, as described previously [22]. The primers for the genes of interest are shown in table 1. The protocol was validated for each gene by checking melting curves for the absence of primer-dimers or other unwanted amplicons. The levels of individual mRNAs were normalized with levels of the housekeeping gene acidic ribosomal phosphoprotein Pθ (Arbp) and the results expressed in arbitrary units.

**Table 1:** Primers used for analysis of gene expression by real time quantitative RT-PCR

**Accession no**.	Gene	Gene name	Forward primer (5'-3')	Reverse primer (5'-3')
NM_017340	ACOX1	acyl-coenzyme A oxidase 1	AGCTGTGCTGAGGAACCTGT	CTGGTGGATGCCTTTGACTT
NM_031559	CPT-1a	carnitine palmitoyltransferase 1a	AAGGTGCTGCTCTCCTACCA	TACCTGGAATCTGTGAGGCC
NM_139192	SCD1	stearoyl-coenzyme A desaturase 1	GATATCCACGACCCCAGCTC	TACCTTATCAGTGCCCTGGG
NM_017332	FAS	fatty acid synthase	CTTTGTGGCCTTCTCCTCTG	GCAGTTTTGTGCTGGTTGAG
NM_199115	Angptl4	angiopoietin-like 4	CAGGCTACCACCCTGTTGAT	TGGACAGAGAAGAAGCCCAT
NM_001024743	UGP2	UDP-glucose pyrophosphorylase 2	GGTTTGCTCGACACCTTCAT	TACGAAGGCAAACTGAGGCT
NM_013098	G6Pase	glucose-6-phosphatase	CTACCTTGCGGCTCACTTTC	GACCTCCTGTGGACTTTGGA
NM_198780	PEPCK	phosphoenolpyruvate carboxykinase	CCCAGGAGTCACCATCACTT	GTGTCCCCCTTGTCTACGAA
NM_012571	GOT1	glutamate oxaloacetate transaminase 1	TCCAAGAACTTCGGGCTCTA	GGAGTGGAAAGGAAACGTGA
NM_022402	Arbp	acidic ribosomal phosphoprotein Pθ	CAGCAGGTGTTTGACAATGG	AAAGGGTCCTGGCTTTGCTC

### Hepatic lipid content and fatty acid oxidation

Cellular lipids were extracted from rat liver using chloroform and methanol (2:1, vol/vol), using the Folch method [23]. The extracts were dried, dissolved and analysed for triacylglycerides using the l-α-glycerol phosphate oxidase kit [24] for the determination of triglycerides (Roche Diagnostics). Samples were analysed in triplicate and the results expressed as μg lipid per mg liver weight. Rates of hepatic β-oxidation was assayed by monitoring the palmitoyl-CoA-dependent reduction of NAD to NADH in sub-fractionated liver homogenates, as described previously [22], Samples were analysed in triplicate and the results expressed as μmol NADH produced per minute and mg protein.

### Hepatic glycogen content

Liver homogenates (10%) were extracted in 80% ethanol to remove glucose. An aliquot of each homogenate was mixed with amyloglucosidase (Roche Applied Science) and incubated at 60°C for 15 minutes to degrade glycogen into glucose residuals. The samples were diluted and incubated with 1 ml of Glucose Assay Reagent (o-Dianisidine Reagent + Glucose Oxidase/Peroxidase Reagent, Sigma-Aldrich) at 37°C for 30 min, followed by the addition of 1 ml 12 N H_2_SO_4 _to stop the reaction. The absorbance of glucose was read at 540 nm. In parallel, different concentrations of rabbit liver glycogen type III (Sigma-Aldrich) were treated as the samples and used as standard curve. Samples were analysed in duplicate and the results determined as μg glycogen per μg protein. The Bradford protein assay was used to measure the concentration of protein (Bio-Rad Laboratories).

### Hepatic glucose output

Rats were anesthetized with an intraperitoneal injection of ketamine (Pfizer AB, Täby, Sweden) at a dose of 60-70 μg/g body weight. Livers were perfused *in situ *for 15 minutes without recirculation in a 37°C cabinet via the portal vein using Krebs-Henseleit bicarbonate buffer, pH 7.4, which was equilibrated with 95% O_2 _and 5% CO_2 _as described previously [25]. No gluconeogenic precursors were present in the buffer. The perfusion pressure was kept constant with a flow rate of 3.0-4.0 ml/min/g liver. Six samples from the inferior caval vein were collected at 2 minute intervals, starting five min after perfusion initiation, and their glucose levels were measured bya Glucometer (YSI 2300 STST PLUS, VWR). Hepatic glucose output was calculated using the mean glucose concentration in relation to flow rate and hepatic dry weight. The wet and dry liver weights are presented together with body weights in table 2. These livers were not used for any other measurements. When liver-derived perfusates were analysed using NMR spectroscopy (as described below), only one out of the six collected fractions was used per liver.

**Table 2:** Animal data related to HGO measurements

	Female	Male	Significant sex-difference
Body weight (g)	205.3 ± 6.6	289.4 ± 11.1	P < 0,0001
Wet liver weight (g)	10.0 ± 0.4	14.3 ± 0.7	P < 0.001
Wet liver weight (% of body weight)	4.9 ± 0.1	4.9 ± 0.1	
Dry liver weight (g)	2.2 ± 0.2	3.1 ± 0.3	P < 0.05
Dry liver weight (% of body weight)	0.9 ± 0.2	1.1 ± 0.1	

### Akt and AMPK phosphorylation

Whole liver cell lysates were obtained by homogenizing 1 g of liver in 3 ml RIPA buffer (50 mM Tris-HCl, pH 7.4, 1% Triton X-100, 150 mM NaCl, 5 mM EDTA, 1 mM PMSF, 1 mM Na_3_VO_4_, 10 mM NaF, 1 μg/ml of aprotinin, leupeptin, and pepstatin), using a polytrone PT-2000 (Kinematica AG), followed by 20 minutes of centrifugation (12 000 g). The resulting supernatants were collected and subjected to protein analyses. The degree of insulin signalling was analysed by measuring the degree of insulin-dependent phosphorylation of Akt. Akt activation was determined by analysing the amount of phosphorylated Akt (p-Akt-Ser473) in relation to total Akt, using commercially available ELISA kits (Biosource). Samples were analysed in triplicate and the results determined as unit p-Akt per ng total Akt. The degree of AMPK phosphorylation was determined by immunoblotting as described before [22], using antibodies detecting p-AMPK-Thr172 or AMPK (1:1000) from Cell signaling. Densitometry analysis was performed using the software Quantity One 4.6.5 Basic (Bio-Rad Laboratories) to compare the amount of phosphorylated AMPK (p-AMPK-Thr172) in relation to total AMPK.

### NMR spectroscopy

Liver perfusate samples were generated as described above, and 500 μl from the third fraction was mixed with 50 μL standard solution of Hexa deutero-4,4-Dimethyl-4-silapentane-1-ammonium trifluoroacetate (DSA), purchased from Onyx Scientific Ltd (Sunderland, United Kingdom), and D_2_O in 5 mm SampleJet NMR tubes (Bruker BioSpin, Rheinstetten, Germany) to a final DSA concentration of 0.36 mM and kept at 6°C until analysis. The NMR spectroscopic measurements were made on a Bruker 600 MHz instrument (Bruker BioSpin, Rheinstetten, Germany) operating at 600.23 MHz, equipped with a 5 mm inverse probe and a SampleJet sample changer. ^1^H-NMR spectra were acquired using a Carr-Purcell-Meiboom-Gill (CPMG) spin-echo sequence to attenuate broad signals arising from macromolecular components [26]. 512 transients were acquired into 64 K data points using a spectral width of 12019 Hz with a spin echo loop time of 76.8 ms and relaxation delay of 2.0 s with a total repetition time of 4.82 s. Suppression of the water resonance was achieved by presaturation during the relaxation delay and the spin-echo loop. The time domain data were processed using a exponential window function with a line broadening factor of 0.3 Hz and zero-filled to yield a real spectrum of 64 K data points in the frequency domain after Fourier transformation. The spectra were manually phase corrected using Topspin 1.3 (Bruker Biospin) and imported into Chenomx NMR Suite 5.1 (Chenomx Inc., Edmonton, Canada) for further analysis. Spectra were baseline corrected and referenced to the DSA peak [27,28]. Metabolite signals were identified and quantified using the metabolite library in the software [29].

### Statistical analysis

Two-way ANOVA was performed to determine whether there were significant effects of sex, fasting or insulin treatment on measured variables or whether there was a significant interaction i) between sex and fasting or ii) between sex and insulin. Subsequently, if the interaction was found to be significant, one-way ANOVA was conducted and multiple comparisons with Fisher's Least Significant Difference (LSD) test was employed to compare i) 4h-fasted males and females and the 12 h fasting effect, or ii) saline-treated males and females and the insulin effect in males and females. For ^1^H-NMR metabolite data with low quantification quality, normality could not be assumed and Friedman's non-parametric test was utilized to determine effect of insulin treatment or sex effect. Where indicated, groups were also compared using Student's t-test. P-values < 0.05 were considered significant.

## Results

### Sex-dependent hepatic gene expression

Whole-genome rat oligo microarrays were used to identify genes with a sex-different expression in rat liver. Out of 27 649 genes printed on the arrays, approximately 3 500 were detected in liver. With a 5% false discovery rate and a cut-off at 1.5-fold difference, 383 (11%) transcripts among these were higher in females, and 399 (11%) transcripts were significantly higher in males. The differentially expressed gene products were grouped into functional categories to enable an overview of sex-differences within metabolic pathways. Results related to lipid and carbohydrate turnover are listed in table 3, and the whole data set is available from the NCBI Gene Expression Omnibus database (GEO; http://www.ncbi.nlm.nih.gov/geo/) using the series entry GSE20601.

**Table 3:** Sex-dependent hepatic expression of gene products from metabolic pathways

**Accession no**.	Gene name	Fold sex-difference	
		Female	Male
	**Fatty acid turnover**		
NM_031561	CD36 molecule	4.89	
NM_144748	acyl-CoA synthetase medium-chain family member 2	4.53	
NM_144750	lysophospholipase, asparaginase homolog	2.47	
NM_053607	acyl-CoA synthetase long-chain family member 5	2.35	
NM_012732	lipase A, lysosomal acid, cholesterol esterase	2.32	
NM_199115	angiopoietin-like protein 4	2.26	
NM_001107793	acyl-CoA synthetase short-chain family member 2	2.18	
NM_017274	glycerol-3-phosphate acyltransferase, mitochondrial	1.98	
NM_001012345	diacylglycerol O-acyltransferase homolog 2	1.91	
XM_227765	microsomal triglyceride transfer protein	1.89	
NM_001004085	carnitine acetyltransferase	1.84	
NM_080576	apolipoprotein A-V	1.71	
XM_215367	1-acylglycerol-3-phosphate O-acyltransferase 3	1.70	
XM_231089	1-acylglycerol-3-phosphate O-acyltransferase 2	1.70	
NM_138882	phospholipase A1 member A	1.69	
NM_012556	fatty acid binding protein 1, liver	1.67	
NM_001006995	acetyl-Coenzyme A acetyltransferase 2	1.60	
NM_031736	solute carrier family 27 (fatty acid transporter), member 2	1.59	
NM_017306	dodecenoyl-coenzyme A delta isomerase	1.52	
NM_134383	ELOVL family member 6		15.64
NM_032082	hydroxyacid oxidase 2, long chain		10.35
NM_139192	stearoyl-CoA desaturase 1		3.60
NM_031841	stearoyl-CoA desaturase 2		3.06
NM_053674	phytanoyl-CoA hydroxylase		2.25
NM_031987	carnitine O-octanoyltransferase		2.16
NM_031559	carnitine palmitoyltransferase 1a		2.10
NM_130433	acetyl-Coenzyme A acyltransferase 2		2.02
NM_001034925	carnitine palmitoyltransferase 1c		1.83
NM_013196	peroxisome proliferator activated receptor alpha		1.67
NM_016986	acetyl-coenzyme A dehydrogenase, medium chain		1.63
NM_017075	acetyl-coenzyme A acetyltransferase 1		1.59
NM_012930	carnitine palmitoyltransferase 2		1.52
	**Retinoic acid turnover**		
NM_017158	cytochrome P450, family 2, subfamily c, polypeptide 7	2.70	
NM_199208	retinol dehydrogenase type II		11.16
NM_145084	retinol saturase		2.58
	**Cholesterol and bile acid turnover**		
NM_012941	cytochrome P450, subfamily 51	2.38	
NM_012690	ATP-binding cassette, sub-family B (MDR/TAP), member 4	2.37	
NM_199115	Angiopoietin-like 4	2.26	
XM_221100	ATP-binding cassette, sub-family A (ABC1), member 8a	2.25	
NM_053754	ATP-binding cassette, sub-family G (WHITE), member 5	2.22	
NM_053642	sterol-C5-desaturase	2.13	
NM_022389	7-dehydrocholesterol reductase	1.74	
NM_012942	cytochrome P450, family 7, subfamily a, polypeptide 1	1.69	
NM_001033694	sterol regulatory element binding factor 2	1.57	
NM_031840	farensyl diphosphate synthase	1.56	
NM_057133	nuclear receptor subfamily 0, group B, member 2		2.05
	**Carbohydrate turnover**		
NM_053551	pyruvate dehydrogenase kinase, isoenzyme 4	2.33	
NM_199118	glucosidase, alpha; acid	1.85	
NM_012565	glucokinase		2.49
NM_001008893	lactate dehydrogenase D		2.49
BC076398	isocitrate dehydrogenase 2 (NADP+), mitochondrial		1.94
NM_012600	malic enzyme 1, NADP(+)-dependent, cytosolic		1.84
NM_017025	lactate dehydrogenase A		1.82
NM_012621	6-phosphofructo-2-kinase/fructose-2,6-biphosphatase 1		1.81
BC084711	UDP-glucose pyrophosphorylase 2		1.81
NM_012879	solute carrier family 2 (facilitated glucose transporter), member 2		1.79
NM_032080	glycogen synthase kinase 3 beta		1.66
	**Amino acid turnover**		
NM_022521	ornithine aminotransferase	1.91	
NM_017159	histidine ammonia lyase	1.69	
NM_022619	solute carrier family 7 (cationic amino acid transporter, y+ system), member 2	1.52	
NM_030850	betaine-homocysteine methyltransferase		1.94
NM_021750	cysteine sulfinic acid decarboxylase		1.83
NM_053818	solute carrier family 6 (neurotransmitter transporter, glycine), member 9		1.82
NM_017084	glycine N-methyltransferase		1.64
NM_053962	serine dehydratase		1.62
NM_012531	catechol-O-methyltransferase		1.61
XM_219785	glycine dehydrogenase (decarboxylating)		1.58
NM_017134	arginase 1		1.56
NM_198731	choline dehydrogenase		1.55

Total RNA was extracted from male and female rat livers and sex-dependent gene products were identified using rat whole-genome oligo arrays. The data were obtained using SAM at a false discovery rate of 5% and are represented as mean fold changes. Functional analysis was made but only genes involved in lipid, carbohydrate and amino acid metabolism are listed here. All data are available from the NCBI Gene Expression Omnibus database http://www.ncbi.nlm.nih.gov/geo/ using the series entry GSE20601.

Among the transcripts of relevance for hepatic lipid and carbohydrate metabolism (listed in table 3), those involved in FA turnover constituted the biggest group. The results point towards a female-predominant capacity for hepatic uptake of long-chain fatty acids, synthesis of TG, and assembly of VLDL particles, in line with previous reports [30,31]. In addition, a male-predominant capacity for mitochondrial and peroxisomal oxidation of FA was revealed. Sex-differences within this group of gene products have been described before, and the results obtained in the present study both confirm and extend previous findings [12]. A clear sex-difference was also observed in the expression of genes encoding proteins of importance for cholesterol and bile acid synthesis, with higher expression in female liver. Furthermore, male rats had higher levels of gene products for glucose uptake and synthesis of glycogen.

As described above, 22% of the genes expressed in rat liver were identified as being sex-dependent (11% being male-predominant and 11% female-predominant), as determined by whole-genome microarrays, but only a fraction of these are listed in table 3 (showing only genes related to lipid and carbohydrate turnover). Among those not shown in the table, α-2u-globulin [32], carbonic anhydrase 3 [33], cyp2c13 [34] and cyp2c11 were male-predominant, whereas alpha-1-B glycoprotein [35], insulin-like growth factor binding protein 1 [36], prolactin receptor [37], HNF-6 [38], cyp2c12 [39] and cyp2c7 [40] were expressed to a greater extent in females. These transcripts have previously been shown to be sex-different in rat liver, substantiating the results obtained in the present study.

**Figure 1:**
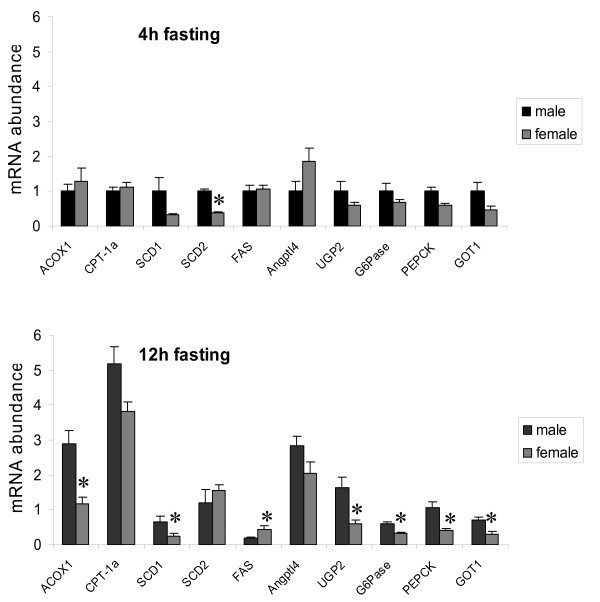
**Sex-dependent mRNA expression of selected genes from metabolic pathways**. mRNA expression levels of selected genes for hepatic amino acid, carbohydrate and lipid metabolism were quantified in male and female rat liver by real-time PCR and normalized to the housekeeping gene Arbp (n = 4 rats/group). Data are represented as means ± SEM, and asterisks indicate significant differences compared to corresponding male group as determined by Student's t-test.

Many gene products from metabolic pathways are regulated in response to starvation. To investigate whether males and females respond differently to this type of metabolic stress, a new set of rats were fasted for either 4 h (absorptive state) or 12 h (post-absorptive state) and compared regarding hepatic gene expression. Ten gene products of relevance for lipid and carbohydrate metabolism were selected for this purpose and measured by RT-PCR. Seven of those were identified from Table 3, whereas glucose-6-phosphatase (G6Pase), acyl-coenzyme A oxidase 1 (ACOX1) and fatty acid synthase (FAS) were selected due to their roles in catalysing important rate-limiting steps in hepatic glucose output, FA oxidation and FA synthesis. Interestingly, most gene products showed a greater sex-difference when the animals were starved (figure 1). Two-way ANOVA revealed a significant sex-fasting interaction on ACOX1 (P < 0.005) and carnitine palmitoyltransferase 1a (CPT-1a, P < 0.05) mRNA levels. Further analysis with Fisher's LDS established that males had higher ACOX1 (P < 0.01) and CPT-1a (P < 0.01) levels than females in the 12h-fasted state. UDP-glucose pyrophosphorylase 2 (UGP2) glutamate oxaloacetate transaminase 1 (GOT1), G6Pase and stearoyl-coenzyme A desaturase 1 (SCD1) were also confirmed as sex-different gene products (P < 0.05).

### Hepatic lipid content, fatty acid oxidation, glycogen content and glucose output

**Figure 2:**
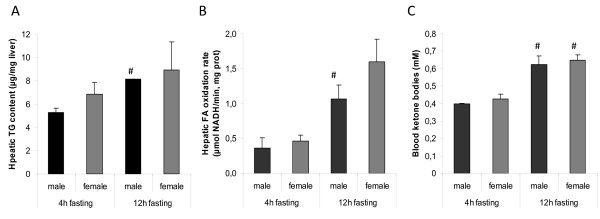
**Effects of fasting on hepatic lipid content, fatty acid oxidation and blood ketone bodies**. (A) Hepatic triglyceride content was measured in lipid extracts from livers of male and female rats (n = 4 rats/group). (B) Rate of fatty acid oxidation was determined in liver homogenates from male and female rats (n = 4 rats/group). (C) Circulating levels of ketone bodies were measured in a drop of blood collected from the tip of the tail (n = 3-5 rats/group). Data are represented as means ± SEM, and asterisks indicate significant differences compared to the corresponding non-starved group as determined by Student's t-test.

**Figure 3:**
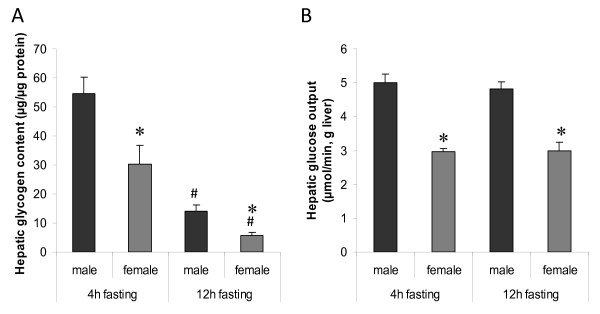
**Sex-dependent hepatic glycogen content and glucose output**. (A) Hepatic glycogen content was quantified in males and females by the amyloglucosidase method (n = 7-9 rats/group). (B) Hepatic glucose output was measured in males and females by in situ liver perfusion (n = 4 rats/group). Levels of glucose production were correlated to corresponding liver dry-weights. Data are represented as means ± SEM, and asterisks indicate significant differences compared to corresponding male (*) or non-starved group (#) as determined by Student's t-test

Hepatic triglyceride (TG) content, FA oxidation rate, and ketone bodies in blood were analysed in 4 and 12h-fasted males and females. Two-way ANOVA revealed that 12h-fasted animals had higher FA oxidation rates (P < 0,001) and ketone levels (P < 0,0001), without any sex-fasting interaction (figure 2). No differences in TG content were observed. However, males had higher levels of hepatic glycogen as compared to females (P < 0,005). The hepatic glycogen stores were reduced in both sexes upon 12 h fasting (P < 0,0001), but the sex-difference was maintained (figure 3A).

**Figure 4:**
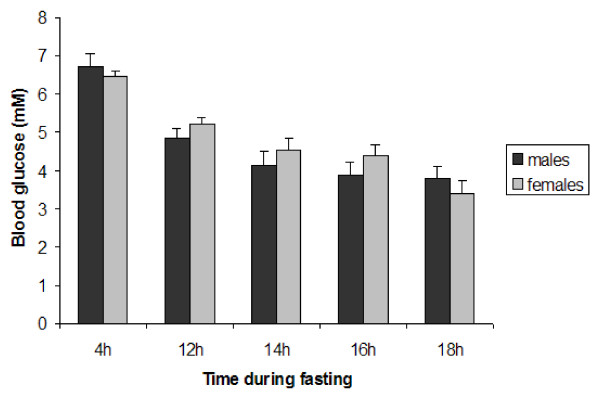
**Blood glucose levels in response to fasting**. Blood glucose levels were measured in a drop of blood collected from the tip of the tail at the indicated time-points after food removal (n = 10 rats/group). Data are represented as means ± SEM.

HGO was determined by *in situ *perfusion of the liver in the absence of gluconeogenic precursors [25]. Interestingly, glucose levels were higher in perfusates collected from male rats (P < 0,0001), but there were no significant differences in HGO between 4 and 12 h fasting (figure 3B). Since the animals were age-matched, body weights and liver weights were different between the sexes. However, the liver weights were the same in males and females when expressed as % of body weight (table 2), suggesting a true sex-difference in the hepatic capacity to release glucose, independent of body weight. When blood glucose levels were determined at different durations of fasting, a significant decrease was observed after 12 h but sex-differences could not be detected at any time point (figure 4). The discrepancy between glucose levels in blood and liver perfusates might be explained by differences in glucose uptake by peripheral tissues.

**Figure 5:**
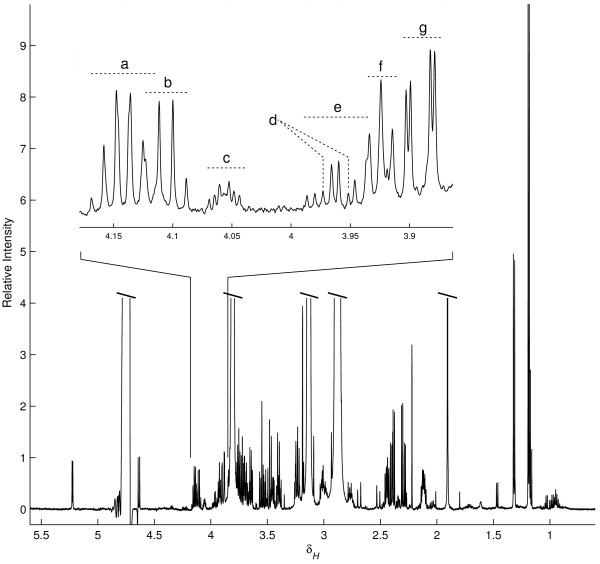
**Typical 600 MHz ^1^H NMR spectrum (δ 0.5-5.6) of a male rat liver perfusate sample**. Resonances from the buffer, HEPES and acetate, as well as the residual water signal were cropped to reveal the more interesting liver-derived signals. The middle expansion shows in detail a small portion of the ppm-axis. Assigned signals in the expanded region are (a) 3-hydroxybutarate, (b) lactate, (c) choline, (d) histidine, (e) serine, (f) HEPES (^13^C-satelite), and (g) glucose.

The sex-different rate of hepatic glucose output reported above was obtained by determining glucose levels in liver perfusates by the glucose oxidase method using a glucose analyzer. To explore the possibility that other low molecular weight compounds might be leaving the liver in a sex-dependent manner, the perfusates were also analysed using ^1^H-NMR spectroscopy. A typical NMR spectrum acquired from rat liver perfusates is shown in figure 5. Apart from metabolites originating from the perfusion buffer, glucose, lactate, glycerol, several amino acids and ketone bodies were among the identified compounds (listed in table 4). Quantitative analysis verified that perfusates generated from male livers contained more glucose, as compared to females (P < 0.01). There were also significantly higher levels of lactate detected in male samples (P < 0.01).

**Table 4:** Liver-derived metabolites identified and quantified by ^1^H-NMR spectroscopy

	Male		Female		
Liver metabolite	Saline	Insulin	Saline	Insulin	Significant ANOVA effects
3-Hydroxybutyrate	162 ± 29.0	85.3 ± 38.8	130 ± 10.7	111 ± 15.7	
Acetoacetate	12.2 ± 3.7	7.3 ± 3.5	13.7 ± 2.4	12.3 ± 0.7	
Alanine	7.0 ± 2.6	2.2 ± 0.5	6.5 ± 1.8	2.9 ± 0.4	Insulin P < 0.05
Choline	7.6 ± 5.2	23 ± 0.9	5.5 ± 1.7	4.6 ± 1.2	
Formate	5.5 ± 1.0	3.9 ± 0.8	6.5 ± 0.7	4.7 ± 0.2^a^	Insulin P < 0.05
Glucose	459 ± 96.1^e, f^	48.8 ± 15.8^e^	146 ± 66.4^f^	27.4 ± 14.9^a^	Interaction P < 0.05
Glutamate	25.3 ± 6.2^a^	5.7 ± 1.3^a^	17.7 ± 2.9	14.7 ± 3.7	Insulin P < 0.05
Glutamine	41.7 ± 26.5	low^d^	19.1 ± 3.7	low	Insulin^g ^P < 0.01
Glycerol	13.9 ± 7.0	4.9 ± 0.6	5.5 ± 0.5	5.0 ± 0.9	
Glycine	12.8 ± 5.1	6.8 ± 3.3	10.5 ± 2.4	8.1 ± 1.7	
Histidine	low	low	2.6 ± 1.1^b^	low	Insulin^g ^P < 0.05
Isoleucine	3.1 ± 0.8	2.5 ± 1.4^a^	2.3 ± 0.2	3.9 ± 0.7	
Lactate	94.6 ± 20.2^e, f^	19.5 ± 2.2^e^	33.0 ± 10.1^f^	27.1 ± 8.5	Interaction P < 0.05
Leucine	6.1 ± 1.3	4.3 ± 2.0^a^	3.8 ± 0.2	7.6 ± 1.3	
Phenylalanine	2.6 ± 0.5^a^	low	1.0 ± 0.3	2.7 ± 0.6^b^	
Propionate	6.7 ± 1.4	2.5 ± 0.8^a^	7.8 ± 2.1	4.6 ± 1.3^a^	Insulin P < 0.05
Serine	11.6 ± 5.2	3.2 ± 1.1^b^	8.7 ± 0.7	11.7 ± 3.2	
Succinate	4.8 ± 2.6	0.8 ± 0.2	1.6 ± 0.5^a^	0.8 ± 0.2	
Tyrosine	low	Low	0.9 ± 0.3^c^	1.6 ± 0.5^c^	
Uridine	low	low	ND	ND	Sex^g ^P < 0.05
Valine	4.7 ± 1.1	3.3 ± 1.6	3.1 ± 0.4	5.3 ± 1.0	

Liver perfusates, generated from 12h-fasted male and female rats treated with saline or insulin (s.c.), were anylsed using ^1^H-NMR spectroscopy. The data represent measured concentrations (μM) of analytes and are presented as means ± SEM (n = 4) together with results of statistical analysis.

^a ^1 measurement below limit of reliable quantification, still used in calculations

^b ^2 measurements below limit of reliable quantification, still used in calculations

^c ^Not detectable in one sample imputed by lowest detected/2

^d ^Low indicates not calculated due to multiple low or ND samples

^e ^Male Saline vs Male Insulin, Fisher's LSD, P < 0.001

^f ^Male Saline vs Female Saline, Fisher's LSD, P < 0.01

^g ^Evaluated with Friedman's test

### Insulin-mediated effects

**Figure 6:**
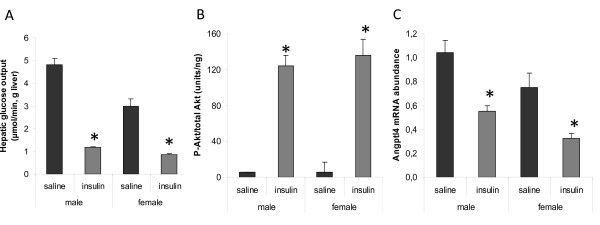
**Effects of insulin on hepatic glucose output, Akt-phosphorylation and Angptl4 mRNA expression in 12h-fasted male and female rats**. (A) Hepatic glucose output was measured in liver perfusates 40 min after saline- or insulin-treatment (n = 4 rats/group). (B) The ratio between phosphorylated (p-Akt-Ser473) and total Akt was determined using ELISA in whole liver cell lysates derived from fasted rats 40 min after saline- or insulin-treatment (n = 6-7 rats/group). (C) Hepatic Angptl4 mRNA levels were quantified by real-time PCR and normalized to the housekeeping gene Arbp (n = 4 rats/group). Data are represented as means ± SEM, and asterisks indicate significant differences compared to corresponding saline-treated group as determined by Student's t-test.

To evaluate the physiological significance of the herein described measurements of hepatic glucose production, the animals were injected (i.p.) with insulin 40 min before initiating liver perfusions. As shown in figure 6A, the rate of glucose output was reduced upon insulin treatment in both male and female livers when compared to saline-injected animals (P < 0.0001). Furthermore, two-way ANOVA revealed a significant sex-insulin interaction on glucose levels (P < 0.005), with a greater response in males. Sex-different effects on HGO were also observed using data obtained by ^1^H-NMR spectroscopy (table 4). However, the extent of insulin-mediated HGO suppression was similar between the sexes when expressed as % of basal glucose levels (glucose analyzer, males 75% and females 71%; ^1^H-NMR spectroscopy, males 89% and females 82%). This indicates that male and female rat livers were equally sensitive to insulin and that the sex-difference in HGO was maintained upon insulin treatment.

Apart from glucose, other liver-derived analytes (lactate, alanine, glutamate, glutamine, histidine, propionate and formate) were also shown to be reduced in perfusates upon insulin treatment (table 4). Two-way ANOVA revealed a significant sex-insulin treatment interaction on lactate levels (P < 0.05). Further analysis with Fisher's LDS established that control males had higher glucose (P < 0.01) and lactate (P < 0.01) than control females and that both glucose and lactate (P < 0.001) decreases with insulin treatment in males.

To test whether male and female livers respond equally well to insulin at the level of insulin receptor signalling, the responses to insulin were compared between male and female rats regarding Akt-phosphorylation (Ser473) and gene expression. No sex-difference could be observed in the degree of Akt-phosphorylation 40 min after insulin treatment, using an ELISA assay (figure 6B). The ratio between phosphorylated and total Akt was increased by the same extent in males (2185%) and females (2427%). Among the gene products from figure 1, angiopoietin-like 4 (Angptl4) showed the most robust response to insulin with a significant reduction in mRNA levels upon this short-term (40 min) insulin treatment. A similar degree of suppression was observed in both sexes (fig 6C, males 47% and females 56%). The effect on blood glucose levels was the same in males (reduced from 6.1+0.3 to 2.5+0.3) and females (reduced from 5.8+0.4 to 2.3+0.4) 40 min after insulin treatment. Furthermore, males and females recovered equally well from insulin-induced hypoglycaemia (data not shown). Although a different dose of insulin might have given different results, this indicates that male and female rats responded equally well to the dose used in this study (5 mU/g body weight).

### Plasma levels of insulin, glucagon and corticosterone

Overall, glycogen levels are increased in response to insulin and decreased by glucagon, whereas the opposite is true for gluconeogenesis. In addition, glucocorticoids influence tissue responses to insulin (inhibiting) and glucagon (stimulating). To find an explanation for the higher glycogen content in male rats, plasma levels of insulin, glucagon and corticosterone were determined. No significant sex- or fasting-related differences in glucagon or insulin were observed (table 5), whereas only females had increased levels of corticosterone in response to 12 h fasting. Importantly, males had higher ratios of insulin to glucagon at both time points. The highest ratio was observed in the absorptive males, and the lowest in the fasted females. This might explain the sex-different levels of hepatic glycogen, and since glycogen serves as a substrate for glucose production it might also explain the male-predominant HGO. It should also be noted that males and females had the same blood glucose levels at these time points (table 5). This together with higher ratios of insulin to glucagon in males indicates that the males were less sensitive to insulin.

**Table 5:** Circulating levels of insulin, glucagon, corticosterone and glucose

	Male	Female	Significant sex-difference
**4 h fasting**			
Insulin (ng/ml)	2.43 ± 0.45	1.56 ± 0.04	
Glucagon (pg/ml)	99.8 ± 6.2	100.9 ± 8.5	
Corticosterone (ng/ml)	495.9 ± 9.9	531.5 ± 22.6	
Insulin-Glucagon ratio	24.3 ± 4.1	16.0 ± 2.0	P < 0.05
Glucose (mM)	6.7 ± 0.3	6.4 ± 0.2	
**12 h fasting**			
Insulin (ng/ml)	3.99 ± 1.75	0.82 ± 0.16	
Glucagon (pg/ml)	275.5 ± 122.4	100.9 ± 2.2	
Corticosterone (ng/ml)	506.1 ± 20.2	587.4 ± 6.9	P < 0.005
Insulin-Glucagon ratio	16.5 ± 2.3	8.0 ± 1.4	P < 0.05
Glucose (mM)	4.8 ± 0.2	5.2 ± 0.2	

Male and female rats were fasted for 4 or 12 h, blood samples were collected and analyzed for insulin, glucagon, corticosterone and glucose. The ratio between insulin and glucagon was calculated. The data are presented as means ± SEM (n = 4) and analyzed using two-way ANOVA and Fisher's LSD test.

### Hepatic AMP kinase

**Figure 7:**
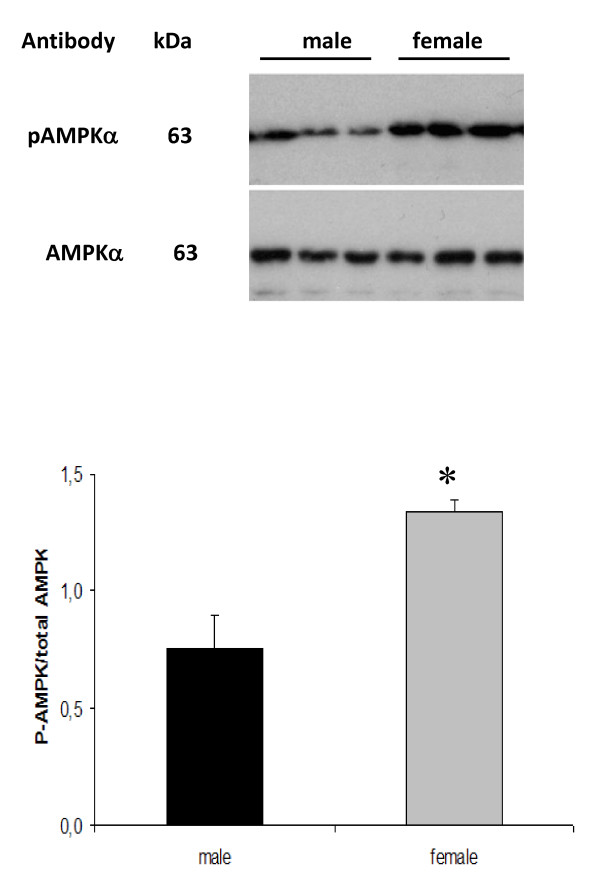
**Sex-dependent degree of hepatic AMP kinase-phosphorylation**. The ratio between phosphorylated (p-AMPK-Thr172) and total AMPK was determined in whole-cell lysates by immunoblotting (upper section) and quantified by densitometry analysis (lower section). Data are represented as means ± SEM, and the asterisk indicates significant difference compared to corresponding male group as determined by Student's t-test.

AMP kinase (AMPK) is an important energy sensor and regulator of cellular fuel metabolism that is under the control of AMP/ATP ratios [41], glycogen content [42] as well as signals depending on hepatocyte receptors for leptin [43] and adiponectin [44]. Activation of this kinase decreases *de novo *lipid synthesis, facilitates FA oxidation and down-regulates genes for gluconeogenesis. To determine whether sex-differences in insulin to glucagon ratios, glycogen levels, HGO rates and transcripts for gluconeogenesis might be related to differences in AMPK activity, we assessed AMPK phosphorylation (Thr172) in male and female livers. As shown in figure 7, the degree of AMPK phosphorylation was higher in female livers, indicating a higher activity level of this kinase in females.

## Discussion

In the present study, we examined whether healthy male and female rats differ regarding hepatic lipid and carbohydrate metabolism. Starting at the level of gene expression by comparing sex-dependent transcript profiles from liver, it was evident that the capacity for different metabolic pathways might differ between males and females. Many genes for the production of TG, cholesterol and VLDL particles were found to be female-predominant, whereas genes for FA oxidation were more expressed in males. In line with this, female rats have previously been shown to have higher rates of hepatic FA uptake [21,30], esterification [21], VLDL-TG formation and output [31] as compared to males. In the present study no differences in hepatic TG content was detected, suggesting that a putative female-predominant capacity regarding TG synthesis was compensated for by a greater VLDL-TG output.

Among the transcripts showing the biggest difference between males and females, CD36 (also known as fatty acid translocase) has been implicated as an important player in the context of hepatic FA uptake. CD36 is increased during situations of increased hepatic lipid content [12,45,46] but reduced during starvation [47], indicating that it might be involved in anabolic actions. Thus, the degree of sex-different CD36 expression depends on the metabolic state of the animal [47]. Similarly, male-predominant responses to 12 h fasting were obtained for ACOX1 and CPT-1a in this study. Taken together, these differences might be part of a sex-different system to handle metabolic alterations, giving rise to secondary sex-differences in hepatic lipid metabolism. It can therefore be speculated that although we could not detect any sex-differences in TG levels, FA oxidation rates or circulating levels of ketone bodies in this study, a different metabolic state with e.g. higher blood levels of non-esterified fatty acids (NEFA) might have generated differences in lipid turnover.

The peroxisome proliferator-activated receptor α (PPARα) is a lipid-activated transcription factor that controls a variety of genes in several pathways of lipid metabolism [48,49]. During situations of increased adipose lipolysis and availability of NEFA in the blood stream, the liver will take up more NEFA which will lead to PPARα activation and increased expression of gene products for FA oxidation [50] and ketogenesis [51]. Male rats have been shown to have higher levels of hepatic PPARα [52] and to be more responsive to peroxisome proliferators than female rats [16-19], including higher rates of FA oxidation. Our finding that starvation induced the PPARα-dependent genes ACOX and CPT-1a in males to a greater extent than in females might thus be explained by higher levels of PPARα in male liver.

An interesting sex-difference related to PPARα and fuel metabolism has been described in mice, where females have much better chances of surviving in a model of defect mitochondrial FA oxidation [53]. When mitochondrial β-oxidation was pharmacologically blocked in mice deficient in PPARα (PPARα^-/-^), TG accumulated in liver and the animals died from hypoglycemia. This happened in 100% of the males but only in 25% of the females. Thus, female mice seem to be less dependent on mitochondrial lipid oxidation. The mechanisms behind these sex differences are not known, but the metabolic phenotype of male PPARα^-/- ^mice was rescued by a 2-week pre-treatment with estrogen [53].

Apart from controlling cellular lipid utilization, PPARα is also an important mediator of gluconeogenesis [54-57]. Gluconeogenesis is fuelled by energy generated through β-oxidation, and fasted PPARα^-/- ^mice develop fatty liver and hypoglycemia [58]. Since PPARα-induced FA oxidation is linked to gluconeogenesis, a higher degree of PPARα activation in males might also explain the male-predominant expression of hepatic G6Pase and PEPCK mRNA in this study. A large extent of the hepatic energy requirement is covered by amino acid oxidation. Hepatic amino acid transferases and deaminases contribute to this through the generation of pyruvate and other glucogenic substrates. A male-predominant expression of GOT1 (glutamate oxaloacetate transaminase 1, also known as aspartate aminotransferase) was observed, which mirrored the mRNA levels of G6Pase and PEPCK. GOT1 catalyzes the transfer of an amino group from an amino acid (glutamate) to a 2-keto-acid to generate a new amino acid and the residual 2-keto-acid of the donor amino acid (alpha-keto-glutarate). Alpha-keto-glutarate is a TCA cycle intermediate and glutamate is one of the most important glucogenic amino acids. Although various mechanisms might underlie the sex-different hepatic glucose output, higher levels of these three important glucogenic enzymes in males are likely to be involved.

^1^H-NMR spectroscopy analysis on rat liver perfusates has to our knowledge not been performed before. Using DSA as internal standard (instead of the more common TSP or DSS) enabled absolute concentrations of metabolites to be determined (20). Using this approach, glucose, lactate, glycerol, propionate, several amino acids (glutamine, glutamate, glycine, serine, alanine, leucine, valine, isoleucine, phenylalanine, histidine and tyrosine) and ketone bodies (3-hydroxybuturate and acetoacetate) were identified as being released from the liver. ^1^H-NMR spectroscopy can be used to measure all kinds of small molecule metabolites simultaneously but is relatively insensitive compared to mass spectrometry-based techniques. Since NMR is close to being a universal detector, the identified metabolites are likely to be the most abundant liver-derived endogenous compounds, at least in 12h-fasted rats. Quantitative analysis verified that perfusates generated from male livers contained more glucose than female perfusates. Liver-derived lactate was also higher in males, and there was a trend towards higher levels of glycerol and the glucogenic amino acids glutamine and glutamate in males. This indicates that the male liver is more active in producing and exporting not only glucose but also metabolites important for glucose production.

A sex-difference in hepatic glucose output has as far as we know not been described before, but there are reports on male-predominant hepatic glycogen content, G6Pase [59] and PEPCK [60] activities. PEPCK activity has further been shown to be reduced by castration or estrogen treatment. Estrogens have also been shown to decrease the expression of PEPCK in virgin female rats [61] as well as G6Pase protein levels and enzyme activity in ob/ob mice [62]. Estrogens have therefore been proposed as an effective antihyperglycemic agent in this model of type 2 diabetes (T2D). Accordingly, estrogen receptor α deficient (ERKO) mice develop fatty liver, have higher fasting blood glucose, plasma insulin levels and impaired glucose tolerance [11]. Since estrogen levels are higher in fertile females than males, estrogen signaling seems to be essential for these hepatic sex-differences. However, other sex-dependent hormones such as growth hormone (GH) are likely to be of similar importance.

The secretion of GH is sexually dimorphic in rats [63] and other species, including humans [64]. In adult male rats, GH is secreted in episodic bursts at 3 to 4 h intervals with low or undetectable levels between peaks, whereas females have a more continuous pattern of secretion. In rodents, biological effects of this sexually differentiated pattern of GH secretion includes sex-differences in body weight gain and longitudinal bone growth [65], but also hepatic steroid metabolism [66]. Using hepatic gene expression profiling, we [12] and others [13] have previously tried to estimate the degree of sex-dependent genes that are controlled by GH. In rats, at least 30% of hepatic sex-differences are explained by the female-specific secretion of GH, through the induction of female-predominat transcripts and suppression of male-predominant. Continuous administration of GH has been shown to increase hepatic expression of SREBP-1c and its downstream target genes [67,68], as well as hepatic triglyceride synthesis and very low-density lipoprotein (VLDL) secretion [69-71]. Furthermore, PPARα which is male-predominant in rat liver has been shown to be suppressed by continuous GH infusion in hypophysectomized [52,72] or old [68] rats and by GH treatment in cultured rat hepatocytes [73]. Taken together, these findings suggest a connection between sex-dependent, GH- and fatty acid-mediated events of relevance for hepatic lipid metabolism.

There is an established link between gluconeogenesis and glycogen synthesis, meaning that a higher rate of glucose production from e.g. amino acids, lactate or glycerol will also lead to a greater capacity to synthesise glycogen. A similar pattern of mRNA expression was observed for UGP2 as for the glucogenic enzymes GOT1, G6Pase and PEPCK, suggesting a common mechanism of regulation. Further experiments are required to determine whether this explains the male-predominant content of glycogen. Another interesting sex-difference was observed regarding plasma levels of insulin and glucagon. Male rats had higher ratios of plasma insulin to glucagon levels, which might also contribute to higher glycogen content in males. In spite of this sex-difference in insulin to glucagon ratios, blood glucose concentrations were the same in male and female rats, which might be interpreted as male rats being less sensitive to insulin. It should also be noted that AMPK was Thr172-phosphorylated to lower degrees in male livers, indicative of higher activity through glucogenic pathways as compared to females. Recent studies have revealed that glycogen can block the activity of AMPK as well as the upstream AMPK kinases LKB1 and CaMKKβ [42]. Whether a cause-effect relationship between higher insulin to glucagon ratios, greater capacity for glycogen synthesis and less AMPK activity exists in male rat livers, and whether this explains the greater HGO in male rat livers observed in this study, have to await further investigations.

The suppressive effect of insulin on hepatic Angptl4 (Angiopoietin-like 4, also known as fasting-induced adipose factor) has to our knowledge not been described before and deserves further studies. Angptl4 is a blood-borne hormone directly involved in regulating glucose homeostasis, lipid metabolism, and insulin sensitivity [74]. Hepatic expression of Angptl4 is increased during starvation [75] and reduced upon short-term high-fat feeding [22]. Data has been presented suggesting that via physical association with plasma lipoproteins [76], Angptl4 acts as a powerful signal to prevent fat storage and stimulate fat mobilization [77]. Thus, insulin might mediate some of its anabolic effects through inhibition of hepatic Angptl4. Angptl4 was one of the female-predominant gene products identified herein, and there was a trend towards higher mRNA levels of Angpl4 in absorptive females. This putative sex-difference disappeared upon starvation due to increased levels in both sexes.

Although we believe that our findings reflect true sex-differences within the liver, we realize that study limitations exist. The significance of the sex differences described here may thus be confounded by differences in e.g. body composition, dietary intake, sensitivity to nutrients, composition and differentiation of the liver, as well as in dynamics of metabolism. However, efforts were made to reduce such confounding factors by recording essential parameters related body and tissue weights and expression analysis of house-keeping genes. Since sex-differences in the liver are related to actions of sex-steroids one may predict that a complete analysis of the metabolome would detect differences in sex-steroids/metabolites. In the present study we did not detect changes in steroids because we used techniques that were not sensitive enough to detect this part of the metabolome.

## Conclusions

It is evident that fuel metabolism differs between male and female animals, but also between men and women [15,78,79]. In the post-absorptive state, plasma concentrations of NEFA are lower whereas glucose and insulin levels are higher in men than in women [80]. Furthermore, men have been shown to utilize less fat and more carbohydrates and amino acids during endurance exercise [81,82]. Data presented in this report are in line with this and lend further support to the picture of males having a greater carbohydrate and amino acid turnover. Our results include higher ratios of insulin to glucagon levels, higher levels of hepatic glycogen, lower degree of hepatic AMPK phosphorylation, higher expression of hepatic gluconeogenic genes and higher hepatic glucose output in healthy male rats. Glycogen storage and release of glucose are major functions of the liver, and the possibility that they might be regulated by sex-dependent mechanisms is intriguing. Sex-differences in hepatic fuel metabolism are probably linked to differences in body composition and physiological demands. Whether these differences are of importance during the development of fatty liver, hepatic insulin resistance and impaired glucose tolerance is not known, but it might be speculated that they contribute to a higher risk of developing T2 D in men during situations of insufficient insulin production.

## Authors' contributions

CG took part in the design of the study, carried out animal experiments, real-time PCR analyses, FA oxidation, glucose, glycogen, AMPK and hormone determinations. KY was responsible for liver perfusions and HGO determinations. EW and JL contributed with NMR spectroscopy and statistical analysis. LC performed microarray analysis and triglyceride determinations. CGG, KB and GN took part in the design of the study and helped to draft the manuscript. PTE participated in design and coordination of the study, took part in animal experiments and drafted the manuscript. All authors read and approved the final manuscript.
